# Atorvastatin prevents *Plasmodium falciparum *cytoadherence and endothelial damage

**DOI:** 10.1186/1475-2875-10-52

**Published:** 2011-02-28

**Authors:** Zacharie Taoufiq, Paco Pino, Nadine N'dilimabaka, Issam Arrouss, Serge Assi, Florent Soubrier, Angelita Rebollo, Dominique Mazier

**Affiliations:** 1 INSERM, UMR S945, Université Pierre et Marie Curie-Paris 6, CHU-Pitié-Salpêtrière, 91 bd de l'Hôpital, 75013 Paris, France; 2 INSERM, UMR S525, Université Pierre et Marie Curie-Paris 6, Paris, France; 3 AP-HP, Groupe hospitalier Pitié-Salpêtrière, Service Parasitologie-Mycologie, Paris, France; 4 Okinawa Institute of Science and Technology, Onna, Okinawa, Japan; 5 Department of Microbiology and Molecular Medicine, University of Geneva, Geneva, Switzerland; 6 Institut Pierre Richet/Institut National de la Sante Publique, Abidjan, Ivory Coast

## Abstract

**Background:**

The adhesion of *Plasmodium falciparum *parasitized red blood cell (PRBC) to human endothelial cells (EC) induces inflammatory processes, coagulation cascades, oxidative stress and apoptosis. These pathological processes are suspected to be responsible for the blood-brain-barrier and other organs' endothelial dysfunctions observed in fatal cases of malaria. Atorvastatin, a drug that belongs to the lowering cholesterol molecule family of statins, has been shown to ameliorate endothelial functions and is widely used in patients with cardiovascular disorders.

**Methods:**

The effect of this compound on PRBC induced endothelial impairments was assessed using endothelial co-culture models.

**Results:**

Atorvastatin pre-treatment of EC was found to reduce the expression of adhesion molecules and *P. falciparum *cytoadherence, to protect cells against PRBC-induced apoptosis and to enhance endothelial monolayer integrity during co-incubation with parasites.

**Conclusions:**

These results might suggest a potential interest use of atorvastatin as a protective treatment to interfere with the pathophysiological cascades leading to severe malaria.

## Background

Malaria remains a major threat to public health with 40% of the world population currently at risk. Every year, an estimated 500 million cases of clinical malaria and at least one million deaths are reported [1,2]. Fatal cases of malaria occur mainly in young children in Africa and consist of an acute neurological syndrome (cerebral malaria), either in isolation or concomitantly with multi-organ failure (pulmonary distress, acute renal failure) [3]. Anti-malarial drugs, such as quinine and artemisinin derivatives are administered intravenously as an emergency treatment. However, although these drugs effectively and rapidly clear parasites from the blood, 15%-20% of patients still die, probably as the result of impaired host responses [4,5]. Such a situation together with the difficulty in developing vaccines highlights the urgent need of novel strategies for complement therapeutics.

The severity of *Plasmodium falciparum *infection depends largely on the ability of parasitized red blood cells (PRBC) to adhere on endothelial cells (EC) and sequester in the capillary network of vital organs (e.g. brain, lungs, kidneys, liver.) Moreover, activation of endothelial cells resulting from the adhesion of infected erythrocytes leads to an overexpression of different mediators, such as adhesion molecules ("hyperadhesion" phenomenon), pro-inflammatory cytokines, coagulation factors and contributes by itself to the pathology [6]. Among endothelial receptors, some are known to interact with PRBC, mainly via PfEMP1 (*P. falciparum *erythrocyte membrane protein 1), a highly polymorphic parasite ligand exported on the infected erythrocyte surface. A number of the cytoadherence receptors have been identified, such as ICAM-1, CD36, VCAM-1 or P-selectin [7]. It was previously shown that *P. falciparum *adhesion to human endothelial cells can specifically trigger proinflammatory gene expression[8-10], oxidative stress [11] and caspases activation [11], further leading to perturbation of the endothelial barrier integrity [12,13]. In addition PRBC adhesion to EC induces redox and rho-kinase dependent EC activation and apoptosis, which can be reversed respectively by the addition of anti-oxidants or fasudil rho-kinase inhibitor [12,14].

Atorvastatin is an oral drug that lowers the level of cholesterol in the blood. All statins, including atorvastatin, prevent the production of cholesterol by blocking the enzyme HMGCoA reductase. However, there is increasing evidence that statins may also exert effects beyond cholesterol lowering. Many of these cholesterol-independent or "pleiotropic" vascular effects of statins appear to involve restoring or improving the endothelial function through increasing the bioavailability of nitric oxide, promoting re-endothelialization, reducing oxidative stress, and inhibiting inflammatory responses [15]. Thus, the endothelium-dependent effects of statins are thought to contribute to many of the beneficial effects of statin therapy in cardiovascular disease [16]. In addition to its lipids lowering effects, atorvastatin has been shown to promote nitric oxide (NO) production by decreasing caveolin-1 expression in EC, regardless of the level of extracellular LDL-cholesterol [17]. Statins retard the initiation of atherosclerosis formation through the improvement of NO bioavailability by both up-regulation of endothelial-nitric oxide synthase (eNOS) mRNA and decrease of superoxide anion O_2_^- ^production in EC [18]. Statins modulate the adhesion cascade at multiple points by targeting both the endothelium and leukocytes and affect cell adhesion by inhibiting chemokine expression (MCP-1) in activated leukocytes and endothelial cells. There is also evidence that statins decrease ICAM-1 expression in stimulated EC and monocytes [19]. The effects of atorvastatin on mature EC are correlated with the activation of the anti-apoptotic Akt pathway, as determined by the phosphorylation of Akt and eNOS [20]. Therefore, activation of Akt represents a mechanism that can account for some of the beneficial side effects of statins.

Given the pleiotropic effects on endothelium of statins in general and atorvastatin in particular, atorvastatin was hypothesized to be useful as a protective drug against *P. falciparum *induced endothelial damages. Primary human lung endothelial cells (HLEC) were used here in co-culture with *P. falciparum*-infected erythrocytes Previous studies have already shown that PRBC adhesion triggers inflammation, oxidative stress and apoptosis within lung endothelial cells [9,11,12,21,22]. Moreover, acute respiratory distress as a complication of malaria infection is rare, but with a vey high rate of mortality [3,23]. The data presented in this manuscript show that, concomitantly with increased expression of Akt within HLEC, atorvastatin reduces adhesion of *P. falciparum*-infected erythrocytes, and as a consequence, prevents the endothelial damages induced by PRBC.

## Methods

### Culture of human endothelial cell

Primary endothelial cells were isolated from human lung (HLEC) after enzymatic digestion and selected using a continuous gradient and immunomagnetic purification technique, as described elsewhere [24]. Endothelial cells of ninth to twelfth passages derived from one batch were used for the experiments. Before use, cells were verified for their expression of ICAM-1, CD36, Von Willebrand factor, VCAM-1, CD31, E/P-selectin and CSA. HLEC were raised in M199 medium (Gibco), supplemented with 10 μg/ml of endothelial cell growth supplement (Upstate, NY) and 10% of fetal calf serum (Biowest), at 37°C with 5% CO_2_, using 8 chamber-Labtek (Nunc International, Napervil), 35 mm glass bottom dishes (MatTek corp.), 96-well plates (Costar), or Transwell insert supports (Corning LifeSciences).

### *Plasmodium falciparum *culture

The *P. falciparum *3D7 clone was used for these experiments. Infected erythrocytes were maintained in culture according to Trager and Jensen's technique in a suspension of erythrocytes in RPMI (Gibco) supplemented with 8.3 g/l of Hepes, 2.1 g/l of NaHCO, 0.1 mg.ml^-1 ^of gentamycin, 2 g/l of dextrose, and 0.4% of Albumax II (Gibco, Invitrogen Corporation) [25]. The 3D7 clone was characterized for adhesion phenotype as previously described [25] and adheres to ICAM-1 and CD36. For each experiment, parasite cultures were enriched in mature forms by Plasmagel floating [26]. Briefly, erythrocytes were harvested from 5 to 10% parasitized cultures and centrifuged 5 minutes at 2000 rpm. Cells were resuspended in Plasmion^® ^and incubated for 20 minutes at 37°C. The upper fraction containing mature trophozoites and schizonts was collected and washed three times in RPM, before adequate adjustment of the hematocrit and parasitemia.

### Real time RT-PCR

HLECs were treated with atorvastatin (Pfizer) for 24 hours, and total RNA was prepared using RNeasy mini kit (Qiagen). RT and quantitative PCR were performed as previously described [27]. Primers used were as follow: ICAM-1: forward 5'-GCAATGTGCAAGAAGATAGCCA-3' reverse 5'-GGGCAAGACCTCAGGTCATGT-3' VCAM-1: forward 5'-GAGTACGCAAACACTTTATGTCAATGT-3', reverse 5'-CTCGTCCTTTCGGGACCG-3', P-selectin forward 5'-AGACTCCCCACCAATGTGTGA-3', reverse 5'-CCACGAGTGTCAGAACAATCCA-3' CD36 forward 5'-TAATGGCACAGATGCAGCCT-3', reverse 5'-ACAGCATAGATGGACCTGCAA-3' HPRT1 forward 5'-AAAGGACCCCACGAAGTGTT-3' reverse 5'-TCAAGGGCATATCCTACAACAA-3'.

### *Plasmodium falciparum *adhesion assay

HLEC were raised in Labtek until confluence. Suspensions of mature pRBC were deposited onto cells and incubated for one hour at 37°C with gentle shaking every 10 minutes. After the incubation, the unbound pRBC were removed and the preparation was fixed for 30 minutes at room temperature with 2% glutaraldehyde, before staining with Giemsa. The number of parasites adhering to 700 HLEC was counted by direct observation with light microscope.

### Nucleosome release assay

The proportion of apoptotic cells was assessed by measuring the intracytoplasmic release of mono- and oligonucleosomes as, using Cell Death Dtection ELISA^® ^(Roche) previously described [11]. Briefly, HLEC were raised in 96-well plate (Costar) until confluence and exposed 24 hours to PRBC (hematocrit 5% parasitemia 50%) or RBC (hematocrit 5%). After 5 PBS washing steps, endothelial cells were lysed and cytoplasms were analysed for their nucleosomes content.

### Endothelial barrier integrity assay

Confluent endothelial monolayer were obtained by seeding 30,000 HLEC on Transwell permeable support (polyester, 3 μm pores, 6.5 mm diameter) and raised in M199 medium supplemented as above during 36 hours. Endothelial monolayers were then exposed during 12 hours to PRBC suspensions at 1% parasitemia and 0.5% of hematocrit. Transwell compartments were then washed three times and cell monolayers on Transwell inserts were transferred to a new plate containing PBS. Evans Blue (0.5 mg/mL) (ICN Biomedicals) was then added in the 'upper compartments'. After 5 min of incubation at 37°C and 5% CO_2_, the 'under compartments' were collected for optic density analysis of diffused Evans Blue (630 nm) with a standard microplate reader (Bio-Tek™ EL311SX)[12].

### Western blot

Cells (1 × 10^6^) were lysed in Laemmli sample buffer and protein extracts were separated by SDS-PAGE, transferred to nitrocellulose, blocked (5% non-fat dry milk) in Tris-buffered saline (TBS: 20 mM Tris-HCl pH 7.5, 150 mM NaCl) plus 0.05% Tween-20 and incubated with the primary antibody over night at 4°C in TBS-0.5% non-fat dry milk. The membrane was washed and incubated with PO-conjugated secondary antibody for two hours at room temperature. Secondary antibodies on western blot membranes were revealed using the ECL system.

### Immunofluorescence and confocal microscopy

HLEC were raised in 35 mm glass bottom dishes, before fixation with 1% paraformaldehyde for 5 min, permeabilized and then incubated with polyclonal anti-Akt antibody (Cell Signaling) for 2 h in PBS 3% BSA at room temperature. FITC-secondary antibody was added and incubated for 1 h at room temperature. After several washing steps, samples were incubated with methanol at -20°C for 10 min, mounted with Vectashield medium and analysed by confocal microscopy.

### Statistical analysis

Differences between groups were analysed for statistical significance using the Games-Howell post-hoc (SPSS software). A *p value *at least less than 0.05 was considered significant.

## Results and discussion

### Atorvastatin prevents *P. falciparum*-induced endothelial adhesion molecules upregulation

**Figure 1:**
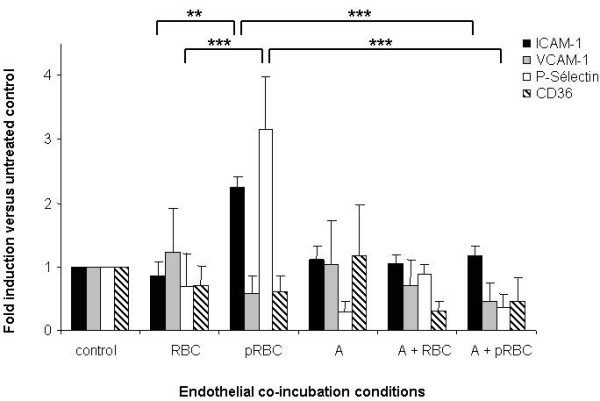
**Effect of atorvastatin on endothelial expression of adhesion molecules**: HLEC were cultured alone or co-cultured with RBC (hematocrit 5%), or PRBC for 4 h (parasitemia 50%, hematocrit 5%) after 24 hours of pretreatment or not with 1 microM of atorvastatin (A, 1 microM). Then, cells were washed and total RNA was isolated. qPCR was done using primers for amplification of human ICAM-1, VCAM-1, P-selectin and CD36. As internal control, we used HPRT. The expression is shown as fold induction compared to control non-treated cells (n = 6), **P < 0.01; *** *P *< 0.005.

In addition to their anti-cholesterol function, statins are also involved in expression regulation of some adhesion molecules in lymphocytes, monocytes and endothelial cells. The role of statin atorvastatin in the control of expression of CD36, ICAM-1, VCAM-1 and P-Selectin, four adhesion molecules that are involved in tethering, rolling and adhesion processes, was analysed in a co-culture of endothelial cells and *P. falciparum *parasitized red blood cells (PRBC). Atorvastatin doses, ranging from 0.01 to 1 microM, showed no toxic effect on human lung endothelial cells (HLEC) *in vitro*, doses ranging from 1.5 microM to 5 microM showed low toxic effects, whereas atorvastatin doses above 6 microM showed mitochondrial toxic effects on HLEC (MTT mitochondrial-based cell viability assay). HLEC were pre-treated 24 hours with 1 microM of atorvastatin before being exposed to PRBC or control RBC during four hours. Total RNA of EC was extracted and adhesion molecules expression was assessed by qPCR. On the one hand, our data show that PRBC increase the expression of endothelial cell (EC) adhesion molecule ICAM-1 and P-selectin. Indeed, PRBC cytoadherence was previously shown to increase cytoadherence itself through increase of EC adhesion molecules expression ('hyperadhesion') [28], contributing to the microcirculation perturbation in severe malaria pathology. More importantly, the data clearly showed that the *P. falciparum*-induced increase of ICAM-1 and P-Selectin expression is suppressed by atorvastatin pre-treatment (Figure 1).

### Atorvastatin decreases *P. falciparum *cytoadherence and *P. falciparum-*induced endothelial apoptosis

**Figure 2:**
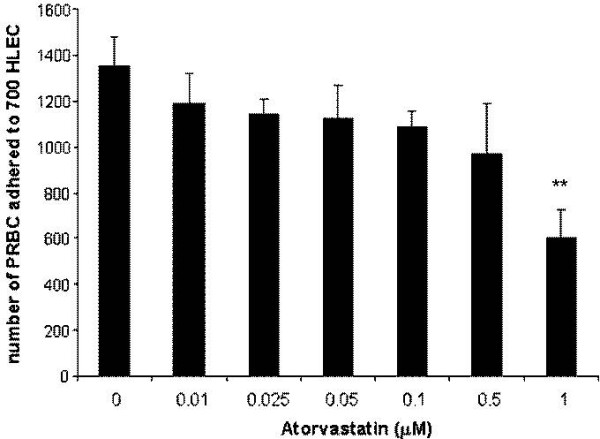
**Atorvastatin decreasing effect on *P. falciparum *endothelial cytoadherence**: HLECs were pre-treated with various doses of atorvastatin ((0.01, 0.025, 0.05, 0.1, 0.5, 1 microM)) for 24 hours before addition of RBCs or PRBCs at a haematocrit of 5% and a parasitemia of 50% for 1 hour. After removal of unbound erythrocytes, cells were stained with Giemsa and the number of adhered pRBC to 700 HLEC was counted. Data are the mean ±SD of pRBC cytodherence (n = 4), **P < 0.01.

Given the effect of atorvastatin endothelial treatment on the expression of adhesion molecules, its effect on PRBC ability to adhere on endothelial cells was analysed (Figure 2). HLEC were pre-incubated 24 hours with various doses of atorvastatin (0.01, 0.025, 0.05, 0.1, 0.5, 1 microM), before being exposed to PRBC ad cytoadherence assay. The data showed that PRBC cytoadherence is significantly decreased by doses that are higher than 0.5 microM of atorvastatin. The cytoadherence decreased to 44% ± 9, for EC pre-incubated with 1 microM of atorvastatin compared to untreated controls. Atorvastatin is thus capable of interfering efficiently with the adhesion of *P. falciparum *infected erythrocytes on endothelial cells.

**Figure 3:**
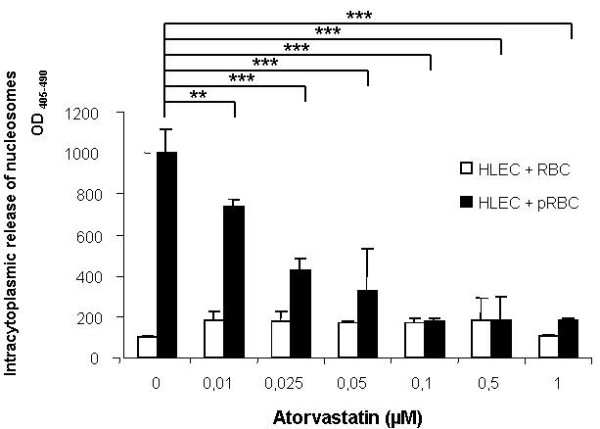
**Effect of atorvastatin on *P. falciparum*-induced endothelial apoptosis**: HLEC cells were pre-treated with or without different concentrations of atorvastatin ((0.01, 0.025, 0.05, 0.1, 0.5, 1 microM)) for 24 h and then co-cultured 4 hours with PRBC (hematocrit 5% parasitemia 50%) or RBC (hematocrit 5%). Intracytoplasmic content of nucleosomes was assessed (n = 4), **P < 0.01; *** *P *< 0.005.

PRBC adhesion was previously shown to specifically trigger endothelial apoptosis [9]. The effect of atorvastatin (0.01-1 microM) on endothelial apoptosis was tested in the co-culture model. HLEC were pre-treated 24 hours with atorvastatin before being exposed for four hours to PRBC or control uninfected RBC. Endothelial apoptosis was then quantitatively assessed using a method to determine HLEC intracytoplasmic content of nucleosomes, a well known late apoptosis marker (Figure 3). The data showed decreasing endothelial apoptosis correlated with increasing doses of atorvastatin pre-treatment. PRBC-induced apoptosis was found completely abolished with doses higher than 0.05 microM.

### Atorvastatin protects endothelial barrier integrity from *P. falciparum-*induced impairments

**Figure 4:**
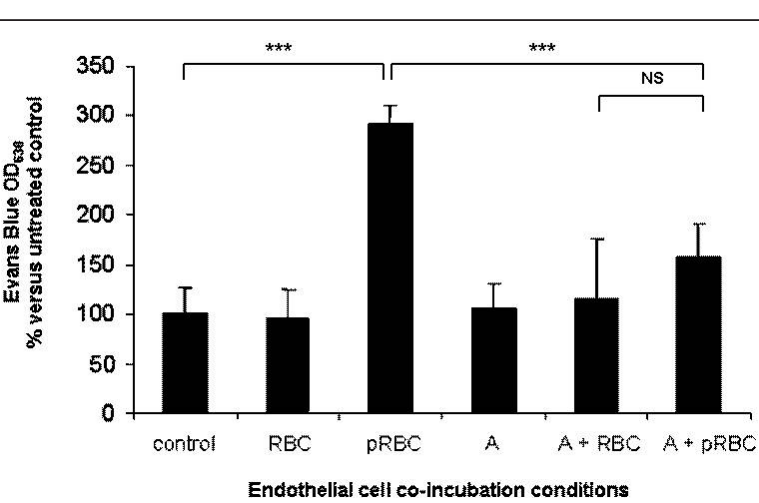
**Protective role of atorvastatin against *P. falciparum*-induced endothelial barrier impairment**: HLEC were pre-treated with 1 microM of atorvastatin for 24 h and then co-cultured with PRBC (hematocrit 0.5%/parasitemia 1%) or RBC (hematocrit 0.5%) for 4 h. The endothelial barrier permeability was estimated by Evan Blue diffusion through an endothelial monolayer. Results are represented related to the OD of the control HLEC cells. A, atorvastatin. (n = 6), *** *P *< 0.005, NS: non significant.

PRBC were previously observed to trigger specific signaling pathway that leads to endothelial barrier permeabilization [12]. Given the effects of atorvastatin pre-treatments on endothelial apoptosis, atorvastatin was tested whether it could have also beneficial properties on endothelial barrier integrity. HLEC were pre-treated with 1 microM of atorvastatin for 24 h and then with PRBC for 4 hours. After washing of endothelial monolayer, the cell barrier integrity was assessed by the standard Evans blue extrusion method. Figure 4 shows that co-culture of PRBC with endothelial cells induces endothelial barrier permeability, compared to unexposed control cells or RBC exposed cells. Pre-treatment of endothelial cells with atorvastatin 1 microM could strongly decrease PRBC-inducced barrier permeabilization. This result suggests that atorvastatin is also very efficient to protect endothelial barrier integrity from *P. falciparum*-induced impairments. However, it should be noted that the relevance of these quantitative data must be restricted to the detection method of standard Evans blue extrusion.

### Atorvastatin increases Akt expression in endothelial cells exposed to *P. falciparum*

**Figure 5:**
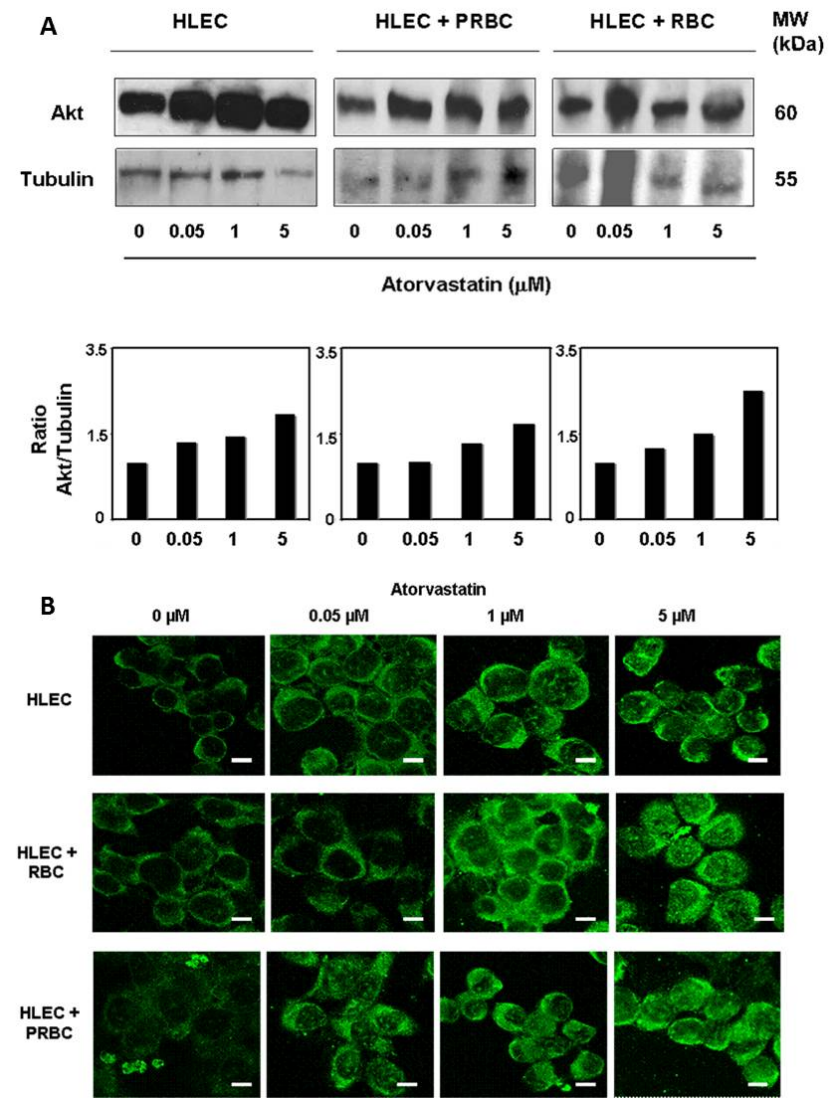
**Atorvastatin promotes endothelial expression of Akt**: **A. Western blot**. HLEC were pre-treated with different concentrations of atorvastatin (0.05, 1 or 5 microM) for 24 h and then cultured or co-cultured with PRBC (at 5% of hematocrit and 50% of parasitemia) or RBC (at 5% of hematocrit) for 4 h. Cells were harvested, washed, protein extracts separated by SDS-PAGE, transferred to nitrocellulose and immunoblotted with anti-Akt and anti-tubulin antibodies, the latter as internal control. Molecular mass of the corresponding proteins is shown. The Western blot data were then quantitatively analysed by densitometry imaging (ratio Akt/Tubulin). Similar results were obtained in three independent experiments. **B. Confocal microscopy**. HLEC were pre-treated with different concentrations of atorvastatin (0.05, 1 or 5 microM) for 24 h and then exposed to PRBC (at 5% of hematocrit and 50% of parasitemia) or RBC (at 5% of hematocrit) for 4 h. Fixed cells were stained with anti-Akt antibody and analysed by confocal microscopy. Similar results were obtained in 3 independent experiments. (Bar: 20 μm).

Among the pleiotropic effects of statins, their role in the activation of cell survival protein kinase B or Akt signaling pathway is well established [29,30]. Given the protective effect of atorvastatin against cell death in our endothelial-PRBC co-culture model, the effect of atorvastatin treatment on endothelial Akt expression was analysed. HLEC were treated 24 hours with 0.05, 1 or 5 microM of atorvastatin. Figure 5A shows Western blot data and its respective quantitative analysis by densitometry imaging. It shows that pre-treatment of increasing doses of atorvastatin up-regulates Akt expression in HLEC. Similarly, Akt expression was up-regulated in co-cultures of HLEC exposed to RBC or PRBC. The effect of atorvastatin on the endothelial up-regulation of Akt expression was confirmed by confocal microscopy imaging analyses (Figure 5B). These data suggest that atorvastatin increases the Akt expression within endothelial cells.

The data presented here demonstrate that atorvastatin can be used to reduce the cytoadherence of *P. falciparum *on endothelial cells, which is one of key events, along with the inflammatory burst, involved in the pathogenesis of human severe malaria cases. Atorvastatin can decrease the expression of adhesion molecules and also prevents the PRBC-induced overexpression of adhesion molecules. Moreover, atorvastatin shows an ability to strongly protect endothelial cell against *P. falciparum*-induced collateral damages, cell apoptosis and endothelial barrier permeabilization.

The observed cytoprotection effect on endothelial cells may mainly be due to the decrease of PRBC adhesion through the down-regulation of adhesion molecules, as PRBC contact was previously shown to specifically trigger pro-inflammatory and pro-apoptotic signaling cascades [9]. Moreover, atorvastatin stimulates Akt expression within HLEC, which is the major actor of anti-apoptotic and endothelial cell survival pathways [29]. Endothelial protection of atorvastatin appears then to be 'pleiotropic' via anti-adhesive, anti-inflammatory and anti-apoptotic synergistic effects. Indeed, PRBC cytoadherence is known to activate many deleterious cascades such as oxidative stress, via mitochondrial ROS (radical oxygen species) production, rho-kinase and NF-kappa B-dependent signaling to induce 'hyperadhesion' phenomenon, endothelial activation and apoptosis [8,11,12,14,28,31]. When phosphorylated, Akt inactivates pro-apoptotic elements such as Bad, preventing mitochondrial ROS release, and caspase 9 [32]. Statins can also increase the bioavailability and the physiological production of endothelial nitric oxide (NO) via Akt phosphorylation and downstream effector endothelial NO-synthase (eNOS), and via the stabilization of eNOS mRNA [30,33]. NO has many crucial functions in vascular endothelium particularly in maintaining permanently anti-inflammatory and anti-adhesive endothelium homeostasis. NO-donors have currently been under investigation in cerebral malaria adjunctive emergency treatments, the efficiency of which remains controversial [34]. Indeed, the deleterious scavenging of NO by ROS has not been considered with antioxidant co-treatment yet. As regard their molecular 'pleiotropic' effects, particularly with the antioxidant and the NO bioavailability promotion, statins may then be highly relevant to 'shield' the endothelium against both PRBC induced impairments and uncontrolled host responses in severe malaria cases.

There is increasing interest of using statins as complementary therapeutic to anti-plasmodial molecules. However, these studies with statins were performed on infected erythrocytes only and have shown interestingly to have a direct inhibitory effect on *P. falciparum *growth *in vitro*, using high doses ranging from 120 to 240 microM [35], from 5 to 40 microM [36] or from 2.5 to 10.8 microM [37]. Atorvastatin was shown to be 10 times more active against *P. falciparum *compared to mevastatin, simvastatin, lovastatin, fluvastatin or pravastatin [36], suggesting that atorvastatin is the best candidate among statins for therapy prospects in malaria treatment. Simvastatin was also used in mouse model of cerebral malaria (C57BL/6 mice infected with *Plasmodium berghei*), but failed to decrease significantly parasitemia or to prevent death [38,39]. Atorvastatin may have a beneficial effect on mice survival, only when administered in combination with artesunate [40]. Indeed, atorvastatin was recently shown to reduce the IC50 of the anti-plasmodial activity of quinine, mefloquine and dihydroartemisinin [41-43]. However, the pathogenesis of cerebral malaria in murine model relies solely on the inflammatory response unlike the pathogenesis of human cerebral malaria. Indeed, the cytoadherence phenomenon does not exist in *P. berghei *mice infections [44,45]. Atorvastatin was used in pre-treatment to specifically evaluate the potential effects on endothelial cells. Endothelial damage is almost universal in severe malaria pathogenesis and statins have proved their efficiency in clinical use for 20 years against cardiovascular diseases [29]. Interestingly, significant endothelial protection effects were obtained with doses as low as 100 nanoM, 500 nanoM or 1 microM.

Although the 'pleiotropic' effects of statins are well documented, the precise molecular mechanism by which the PI3K/Akt/eNOS pathway is activated remains unknown [29,46]. Since the cholesterol lowering property of statins by itself ameliorates the endothelial function, statins beneficial effects were for long attributed to the inhibition of HMG-CoA reductase and the cholesterol synthesis pathway. However, the endothelial function improvement was shown to occur earlier than cholesterol lowering and independently from the cholesterol rate in blood serum [47,48]. Statins are inhibiting the synthesis of mevalonate, the second biochemical reaction in the cholesterol synthesis pathway. Mevalonate is also an important precursor of isoprenoid intermediates such as farnesyl pyrophosphate (FPP) or geranylgeranyl pyrophosphate (GGPP). Isoprenoids serve as cell surface inner membrane-anchoring molecules to functionalize Rho-GTPases, such as Rho (GGPP) and Ras (FPP) [49,50], key signaling molecules, after geranylation and farnesylation post-translational modifications. Rho-GTPases act as molecular switches (active and inactive when bound to GTP and GDP, respectively), and are the first intermediates of the intracellular signaling engagement of these receptors with the downstream effector Rho-kinase. We have previously demonstrated that *P. falciparum*-induced endothelial collateral damages are precisely dependent on Rho-kinase signaling [12]. Rho-kinase pathway by many aspects is antagonist to PI3K/Akt/eNOS endothelial cell survival pathway [46]. In fact, strong endothelial protective effects, obtained with atorvastatin against *P. falciparum*, may likely be related to both rho-kinase pathway inhibition and Akt cell survival pathway promotion. However, it was previously reported that the rho-kinase inhibition by fasudil could not decrease significantly the number of parasites and *P. falciparum *cytoadherence [12,51].

## Conclusions

In conclusion, data from this report demonstrate that HMG-CoA reductase inhibitor atorvastatin prevents *P. faciparum *cytoadherence and confers the ability to the endothelium to resist against consequent cellular damages. In conclusion, this study suggests that atorvastatin may be a good candidate for further studies or clinical trials on the use of statins in malaria treatment. Moreover, the safety of statins on malaria treatment needs to be addressed, since these molecules were not originally designed for these therapeutic approaches.

## List of abbreviations

EC: endothelial cell; RBC: red blood cells; PRBC: parasitized red blood cells; HLEC: human lung endothelial cells.

## Competing interests

The authors declare that they have no competing interests.

## Authors' contributions

ZT contributed to write the manuscript, to design and to conduct the experiments. PP contributed to design the experiments. NN, IA and SA contributed to perform some experiments. FS made substantial constructive advice in the initial design of the project. AR and DM contributed to write the manuscript and to design the experiments. All authors have read and approved the final version of the manuscript.

## References

[B1] Snow RW Guerra CA Noor AM Myint HY Hay SI The global distribution of clinical episodes of *Plasmodium falciparum *malaria Nature 2005 434 214 217 10.1038/nature03342 15759000 PMC3128492

[B2] UNICEF/WHO World Malaria Report 2009 2009 http://www.whoint/malaria/world_malaria_report_2009

[B3] Marsh K Forster D Waruiru C Mwangi I Winstanley M Marsh V Newton C Winstanley P Warn P Peshu N Pasvol G Snow R Indicators of life-threatening malaria in African children N Engl J Med 1995 332 1399 1404 10.1056/NEJM199505253322102 7723795

[B4] Dondorp A Nosten F Stepniewska K Day N White N Artesunate versus quinine for treatment of severe falciparum malaria: a randomised trial Lancet 2005 366 717 725 10.1016/S0140-6736(05)67176-0 16125588

[B5] Idro R Jenkins NE Newton CR Pathogenesis, clinical features, and neurological outcome of cerebral malaria Lancet Neurol 2005 4 827 840 10.1016/S1474-4422(05)70247-7 16297841

[B6] van der Heyde HC Nolan J Combes V Gramaglia I Grau GE A unified hypothesis for the genesis of cerebral malaria: sequestration, inflammation and hemostasis leading to microcirculatory dysfunction Trends Parasitol 2006 22 503 508 10.1016/j.pt.2006.09.002 16979941

[B7] Ho M White NJ Molecular mechanisms of cytoadherence in malaria Am J Physiol 1999 276 1231 1242 10.1152/ajpcell.1999.276.6.C1231 10362584

[B8] Jenkins N Wu Y Chakravorty S Kai O Marsh K Craig A *Plasmodium falciparum *intercellular adhesion molecule-1-based cytoadherence-related signaling in human endothelial cells J Infect Dis 2007 196 321 327 10.1086/518795 17570121 PMC1934551

[B9] Pino P Vouldoukis I Kolb JP Mahmoudi N Desportes-Livage I Bricaire F Danis M Dugas B Mazier D *Plasmodium falciparum*--infected erythrocyte adhesion induces caspase activation and apoptosis in human endothelial cells J Infect Dis 2003 187 1283 1290 10.1086/373992 12696008

[B10] Tripathi AK Sha W Shulaev V Stins MF Sullivan DJ Jr *Plasmodium falciparum*-infected erythrocytes induce NF-kappaB regulated inflammatory pathways in human cerebral endothelium Blood 2009 114 4243 4252 10.1182/blood-2009-06-226415 19713460 PMC2925626

[B11] Pino P Vouldoukis I Dugas N Hassani-Loppion G Dugas B Mazier D Redox-dependent apoptosis in human endothelial cells after adhesion of *Plasmodium falciparum*-infected erythrocytes Ann N Y Acad Sci 2003 1010 582 586 10.1196/annals.1299.109 15033796

[B12] Taoufiq Z Gay F Balvanyos J Ciceron L Tefit M Lechat P Mazier D Rho kinase inhibition in severe malaria: thwarting parasite-induced collateral damage to endothelia J Infect Dis 2008 197 1062 1073 10.1086/528988 18419473

[B13] Tripathi AK Sullivan DJ Stins MF *Plasmodium falciparum*-infected erythrocytes decrease the integrity of human blood-brain barrier endothelial cell monolayers J Infect Dis 2007 195 942 950 10.1086/512083 17330783

[B14] Taoufiq Z Pino P Dugas N Conti M Tefit M Mazier D Vouldoukis I Transient supplementation of superoxide dismutase protects endothelial cells against *Plasmodium falciparum*-induced oxidative stress Mol Biochem Parasitol 2006 150 166 173 10.1016/j.molbiopara.2006.07.008 16930739

[B15] Laufs U Beyond lipid-lowering: effects of statins on endothelial nitric oxide Eur J Clin Pharmacol 2003 58 719 731 12634978 10.1007/s00228-002-0556-0

[B16] Rosenson RS Pluripotential mechanisms of cardioprotection with HMG-CoA reductase inhibitor therapy Am J Cardiovasc Drugs 2001 1 411 420 10.2165/00129784-200101060-00001 14728000

[B17] Brouet A Sonveaux P Dessy C Moniotte S Balligand JL Feron O Hsp90 and caveolin are key targets for the proangiogenic nitric oxide-mediated effects of statins Circ Res 2001 89 866 873 10.1161/hh2201.100319 11701613

[B18] Sumi D Hayashi T Thakur NK Jayachandran M Asai Y Kano H Matsui H Iguchi A A HMG-CoA reductase inhibitor possesses a potent anti-atherosclerotic effect other than serum lipid lowering effects--the relevance of endothelial nitric oxide synthase and superoxide anion scavenging action Atherosclerosis 2001 155 347 357 10.1016/S0021-9150(00)00597-9 11254905

[B19] Romano M Diomede L Sironi M Massimiliano L Sottocorno M Polentarutti N Guglielmotti A Albani D Bruno A Fruscella P Salmona M Vecchi A Pinza M Mantovani M Inhibition of monocyte chemotactic protein-1 synthesis by statins Lab Invest 2000 80 1095 1100 10.1038/labinvest.3780115 10908155

[B20] Urbich C Dernbach E Zeiher AM Dimmeler S Double-edged role of statins in angiogenesis signaling Circ Res 2002 90 737 744 10.1161/01.RES.0000014081.30867.F8 11934843

[B21] Siau A Toure FS Ouwe-Missi-Oukem-Boyer O Ciceron L Mahmoudi N Vaquero C Froissard P Bisvigou U Bisser S Coppee JY Bischoff E David PH Mazier D Whole-transcriptome analysis of *Plasmodium falciparum *field isolates: identification of new pathogenicity factors J Infect Dis 2007 196 1603 1612 10.1086/522012 18008243

[B22] Zang-Edou ES Bisvigou U Taoufiq Z Lekoulou F Lekana-Douki JB Traore Y Mazier D Toure-Ndouo FS Inhibition of *Plasmodium falciparum *field isolates-mediated endothelial cell apoptosis by Fasudil: therapeutic implications for severe malaria PLoS One 2010 5 e13221 10.1371/journal.pone.0013221 20949056 PMC2951358

[B23] Mishra SK Mohanty S Mohanty A Das BS Management of severe and complicated malaria J Postgrad Med 2006 52 281 287 17102547

[B24] Muanza K Gay F Behr C Scherf A Primary culture of human lung microvessel endothelial cells: a useful in vitro model for studying *Plasmodium falciparum*-infected erythrocyte cytoadherence Res Immunol 1996 147 149 163 10.1016/0923-2494(96)83167-1 8817744

[B25] Trager W Cultivation of malaria parasites Methods Cell Biol 1994 45 7 26 full_text 7707996 10.1016/s0091-679x(08)61844-0

[B26] Goodyer ID Johnson J Eisenthal R Hayes DJ Purification of mature-stage *Plasmodium falciparum *by gelatine flotation Ann Trop Med Parasitol 1994 88 209 211 8067816 10.1080/00034983.1994.11812859

[B27] Brun M Bourdoulous S Couraud PO Elion J Krishnamoorthy R Lapoumeroulie C Hydroxyurea downregulates endothelin-1 gene expression and upregulates ICAM-1 gene expression in cultured human endothelial cells Pharmacogenomics J 2003 3 215 226 10.1038/sj.tpj.6500176 12931135

[B28] Tripathi AK Sullivan DJ Stins MF *Plasmodium falciparum*-infected erythrocytes increase intercellular adhesion molecule 1 expression on brain endothelium through NF-kappaB Infect Immun 2006 74 3262 3270 10.1128/IAI.01625-05 16714553 PMC1479273

[B29] Mason JC Statins and their role in vascular protection Clin Sci (Lond) 2003 105 251 266 10.1042/CS20030148 12793855

[B30] Wolfrum S Jensen KS Liao JK Endothelium-dependent effects of statins Arterioscler Thromb Vasc Biol 2003 23 729 736 10.1161/01.ATV.0000063385.12476.A7 12615672

[B31] Schofield L Novakovic S Gerold P Schwarz RT McConville MJ Tachado SD Glycosylphosphatidylinositol toxin of Plasmodium up-regulates intercellular adhesion molecule-1, vascular cell adhesion molecule-1, and E-selectin expression in vascular endothelial cells and increases leukocyte and parasite cytoadherence via tyrosine kinase-dependent signal transduction J Immunol 1996 156 1886 1896 8596041

[B32] Datta SR Dudek H Tao X Masters S Fu H Gotoh Y Greenberg ME Akt phosphorylation of BAD couples survival signals to the cell-intrinsic death machinery Cell 1997 91 231 241 10.1016/S0092-8674(00)80405-5 9346240

[B33] Endres M Laufs U Liao JK Moskowitz MA Targeting eNOS for stroke protection Trends Neurosci 2004 27 283 289 10.1016/j.tins.2004.03.009 15111011

[B34] Sobolewski P Gramaglia I Frangos J Intaglietta M van der Heyde HC Nitric oxide bioavailability in malaria Trends Parasitol 2005 21 9 415 422 10.1016/j.pt.2005.07.002 16039159

[B35] Couto AS Kimura EA Peres VJ Uhrig ML Katzin AM Active isoprenoid pathway in the intra-erythrocytic stages of *Plasmodium falciparum*: presence of dolichols of 11 and 12 isoprene units Biochem J 1999 341 629 637 10.1042/0264-6021:3410629 10417326 PMC1220400

[B36] Pradines B Torrentino-Madamet M Fontaine A Henry M Baret E Mosnier J Briolant S Fusai T Rogier C Atorvastatin is 10-fold more active in vitro than other statins against *Plasmodium falciparum* Antimicrob Agents Chemother 2007 51 2654 2655 10.1128/AAC.01330-06 17502414 PMC1913261

[B37] Parquet V Briolant S Torrentino-Madamet M Henry M Almeras L Amalvict R Baret E Fusai T Rogier C Pradines B Atorvastatin is a promising partner for antimalarial drugs in treatment of *Plasmodium falciparum *malaria Antimicrob Agents Chemother 2009 53 2248 2252 10.1128/AAC.01462-08 19307369 PMC2687225

[B38] Helmers AJ Gowda DC Kain KC Liles WC Statins fail to improve outcome in experimental cerebral malaria and potentiate Toll-like receptor-mediated cytokine production by murine macrophages Am J Trop Med Hyg 2009 81 631 637 10.4269/ajtmh.2009.09-0204 19815878

[B39] Kobbe R Schreiber N May J Jacobs T Simvastatin treatment shows no effect on the incidence of cerebral malaria or parasitemia during experimental malaria Antimicrob Agents Chemother 2008 52 1583 1584 10.1128/AAC.01428-07 18268089 PMC2292537

[B40] Bienvenu AL Picot S Statins alone are ineffective in cerebral malaria but potentiate artesunate Antimicrob Agents Chemother 2008 52 4203 4204 10.1128/AAC.00513-08 18779350 PMC2573154

[B41] Parquet V Henry M Wurtz N Dormoi J Briolant S Gil M Baret E Amalvict R Rogier C Pradines B Atorvastatin as a potential anti-malarial drug: in vitro synergy in combinational therapy with quinine against *Plasmodium falciparum* Malar J 2010 9 139 10.1186/1475-2875-9-139 20497586 PMC2882376

[B42] Savini H Souraud JB Briolant S Baret E Amalvict R Rogier C Pradines B Atorvastatin as a potential antimalarial drug: in vitro synergy in combinational therapy with dihydroartemisinin Antimicrob Agents Chemother 2010 54 966 967 10.1128/AAC.01006-09 19949060 PMC2812141

[B43] Wurtz N Briolant S Gil M Parquet V Henry M Baret E Amalvict R Almeras L Rogier C Pradines B Synergy of mefloquine activity with atorvastatin, but not chloroquine and monodesethylamodiaquine, and association with the pfmdr1 gene J Antimicrob Chemother 2010 65 1387 1394 10.1093/jac/dkq173 20501488

[B44] Lackner P Beer R Helbok R Broessner G Engelhardt K Brenneis C Schmutzhard E Pfaller K Scanning electron microscopy of the neuropathology of murine cerebral malaria Malar J 2006 5 116 10.1186/1475-2875-5-116 17125519 PMC1676017

[B45] White NJ Turner GD Medana IM Dondorp AM Day NP The murine cerebral malaria phenomenon Trends Parasitol 2009 26 11 15 10.1016/j.pt.2009.10.007 19932638 PMC2807032

[B46] Zhou Q Liao JK Statins and cardiovascular diseases: from cholesterol lowering to pleiotropy Curr Pharm Des 2009 15 467 478 10.2174/138161209787315684 19199975 PMC2896785

[B47] MRC/BHF MRC/BHF Heart Protection Study of cholesterol lowering with simvastatin in 20,536 high-risk individuals: a randomised placebo-controlled trial Lancet 2002 360 7 22 10.1016/S0140-6736(02)09327-3 12114036

[B48] O'Driscoll G Green D Taylor RR Simvastatin, an HMG-coenzyme A reductase inhibitor, improves endothelial function within 1 month Circulation 1997 95 1126 1131 9054840 10.1161/01.cir.95.5.1126

[B49] Goldstein JL Brown MS Regulation of the mevalonate pathway Nature 1990 343 425 430 10.1038/343425a0 1967820

[B50] Van Aelst L D'Souza-Schorey C Rho GTPases and signaling networks Genes Dev 1997 11 2295 2322 10.1101/gad.11.18.2295 9308960

[B51] Waknine-Grinberg JH McQuillan JA Hunt N Ginsburg H Golenser J Modulation of cerebral malaria by fasudil and other immune-modifying compounds Exp Parasitol 2010 125 141 146 10.1016/j.exppara.2010.01.005 20093114

